# Heat shock transcription factors in development and disease

**DOI:** 10.1242/dmm.052700

**Published:** 2026-03-30

**Authors:** Roger S. Smith, Marc L. Mendillo

**Affiliations:** ^1^ Department of Biochemistry and Molecular Genetics, Northwestern University Feinberg School of Medicine, Chicago, IL 60611, USA; ^2^ Simpson Querrey Institute for Epigenetics, Northwestern University Feinberg School of Medicine, Chicago, IL 60611, USA; ^3^ Robert H. Lurie Comprehensive Cancer Center, Northwestern University Feinberg School of Medicine, Chicago, IL 60611, USA; ^4^ Medical Scientist Training Program, Northwestern University Feinberg School of Medicine, Chicago, IL 60611, USA

**Keywords:** Transcription, Proteostasis, Cancer, Neurodegenerative disease, Development

## Abstract

The heat shock response is a highly conserved cellular defense mechanism against proteotoxic stress, characterized by the induction of heat shock proteins (HSPs) that function as molecular chaperones to maintain protein homeostasis. Central to this response are the heat shock transcription factors (HSFs), which regulate the expression of HSPs. This Review explores the structural and functional relationships of the mammalian HSF family, including HSF1, HSF2, HSF4 and HSF5. We highlight HSF gene expression and function during organismal development and details of HSFs involvement in neurodegenerative diseases, in which they mitigate/counteract protein aggregation and promote neuronal survival, and in cancer, in which they support tumor growth and metastasis. We also examine the interplay between different HSFs and their context-dependent functions, emphasizing their relevance as potential targets for therapeutic intervention. Understanding the diverse roles of these factors is essential for advancing our knowledge of physiological regulation, and for developing targeted therapies for a broad range of diseases.

## Teaser

**Summary:** This Review examines the mammalian heat shock transcription factor family (HSF1, HSF2, HSF4 and HSF5), summarizing their roles in development, neurodegenerative disease and cancer, and their emerging therapeutic potential.

## Introduction

A coordinated cellular response to elevated temperature can be found universally across many different species (Lindquist, 1986). This heat shock response (HSR) results in the increased expression of heat shock proteins (HSPs), which serve as molecular chaperones to restore protein homeostasis, or proteostasis, in the cell (Akerfelt et al., 2010a; Gomez-Pastor et al., 2018; Lindquist and Craig, 1988; Wu, 1995). The HSR represents one of the most evolutionarily conserved transcription regulatory systems in biology, present in virtually all living organisms, from bacteria to humans. The universal presence of these stress response systems across the tree of life and their significant genetic and functional conservation highlight the profound biological significance of this response pathway.

The HSR was first observed as puffs induced in the chromosomes of the fruit fly, *Drosophila brusckii*, after treatment with heat, dinitrophenol or sodium salicylate (Ritossa, 1962). These chromosome puffs formed as a result of rapid, robust synthesis of RNA and proteins constituting the first molecular descriptions of the HSR (Tissieres et al., 1974). The rapid, readily induced nature of the HSR made it a model system for transcription induction and regulation (Guertin and Lis, 2010; Lindquist, 1986; Mahat et al., 2016; Morimoto et al., 1992; Vihervaara et al., 2017, 2018). In 1984, two groups identified the first evidence for a transcriptional regulator of this response, the heat shock transcription factor (HSF), by studying DNA–protein interactions in heat-shocked *Drosophila* nuclear extracts (Parker and Topol, 1984; Wu, 1984). These works demonstrated the binding of a protein, the activity of which increased with heat, to a consensus sequence of DNA. This canonical heat shock element (HSE), consisting of alternating, inverted repeats of *nGAAn* DNA sequences, where *n* denotes any DNA base, is located upstream of HSP-encoding genes to promote their expression (Parker and Topol, 1984; Wu, 1984). Subsequent studies revealed that invertebrates and ancestral organisms have a single, essential HSF, whereas vertebrates and plants have evolved more complex HSF gene families (Fujimoto and Nakai, 2010).

As a family of transcription factors with broad impact on cellular and organismal biology, HSFs present an opportunity to understand relationships between transcription factor family form and function. Modern genomic approaches have rapidly advanced, enabling more thorough analysis of protein function and relationships in disease-relevant contexts. Here, we review recent work on HSFs that demonstrates complex interactions of HSF family members, which appear tuned to specific biological contexts. Understanding the cell type- or disease state-specific functions of HSFs, operating alone or in concert with HSF family members, will inform our understanding of human health and disease. We also aim to review the structure of HSF family member proteins and its relationship to protein function, with an emphasis on comparisons between HSF family members. Beyond canonical heat shock systems, we explore HSF biology during development, in neurodegenerative disease and in cancer biology. Furthermore, we summarize the latest efforts to target these proteins for novel therapeutics. We conclude by highlighting opportunities for future research to build on decades of advances in this family of transcription factors, which are deeply rooted across the tree of life.

## Functional protein domains within HSFs

**Fig. 1.**
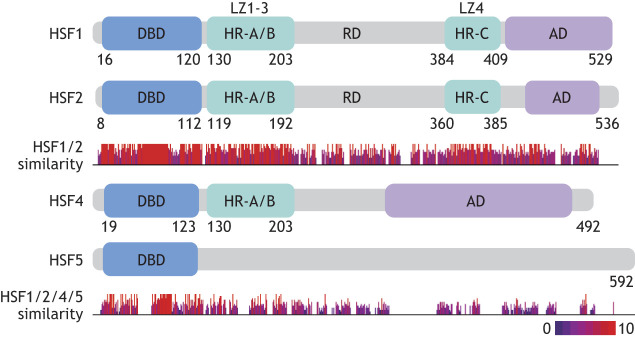
**Domain architecture of the heat shock transcription factor (HSF) family of transcription factors.** All somatic HSF proteins contain an amino-terminal, winged helix-turn-helix DNA-binding domain (DBD). Downstream of the HSF1, HSF2 and HSF4 DBDs are heptad repeat (HR) regions (HR-A/B) that form the leucine zipper (LZ) domains, with LZ1-3 required for HSF oligomerization. A third HR region (HR-C), which forms LZ4, suppresses HSF activity through intramolecular interactions with LZ1-3 in HSF1 and HSF2. Transcriptional activity for some HSFs requires the activation domain (AD). Sequence similarity was determined using Clustal Omega Multiple Sequence Alignment with protein amino acid sequences obtained from Ensembl. Sequence similarity is scored from 0 to 10, as indicated by the height and color of the bars under the respective HSF diagrams.

The human genome encodes four HSFs on somatic chromosomes: HSF1, HSF2, HSF4 and HSF5. Additional genes that encode HSF paralogs (genes that originate from a gene duplication event) exist on the sex chromosomes (*HSFX1-4* and *HSFY1-8*), although evidence of their expression is scant, and their function is underexplored compared to that of other HSF family members (Hastbacka et al., 2025). Each of the somatic HSFs – HSF1, HSF2, HSF4 and HSF5 – contains a highly conserved N-terminal DNA-binding domain (DBD) (Fig. 1). This conservation allows them to bind similar DNA sequences, yet their functional roles diverge owing to differences in other domains and regulatory mechanisms.

The DBD of HSFs is characterized by a winged helix-turn-helix motif. This structure facilitates the binding of HSFs to DNA by allowing specific interactions with the major groove of the DNA helix. The co-crystal structures of human HSF1 and HSF2 trimers bound to DNA reveal a triangular arrangement of the three DBDs, which is crucial for the recognition and binding of HSEs by the complex (Feng et al., 2021; Jaeger et al., 2016). The wing domain contributes to the stability and specificity of DNA binding by facilitating protein–protein interactions within the trimer. HSF2 harbors functional domains that are highly similar to those found in its paralog HSF1, including a DBD, heptad repeat (HR)-A/B and HR-C (Fig. 1) (Fujimoto and Nakai, 2010; Gomez-Pastor et al., 2018), which allow HSF2 to bind the consensus HSE originally defined for HSF1 (Gomez-Pastor et al., 2018). Crystal structures of human HSF1 and HSF2 proteins bound to DNA reveal highly similar HSF–DNA binding interactions  (Jaeger et al., 2016; Neudegger et al., 2016), yet notable differences in the flexibility of the wing domain and in the DNA conformational changes induced by binding (Feng et al., 2021). Moreover, when bound to DNA, HSF1 and HSF2 expose biochemically distinct protein sequences that may serve as substrates for paralog-specific regulation through post-translational modifications or recruitment of other proteins, despite nearly identical DNA binding (Gomez-Pastor et al., 2018; Jaeger et al., 2016). In addition to the canonical HSE, HSF1 and HSF2 can bind to non-canonical HSF binding sites, which have been reported to play significant roles in gene regulation (Himanen et al., 2022). These non-canonical HSEs contain mismatches at certain positions within the consensus sequence but still maintain functional binding affinity (Jaeger et al., 2014). This variability allows for a broader range of regulatory possibilities and fine-tuning of gene expression in response to different stress conditions (Li et al., 2016; Zhao et al., 2020). Owing to higher similarity of the DBD of HSF4 to that of HSF1 and HSF2, it also binds canonical HSEs originally described for HSF1. Indeed, HSF4 DBD crystal structures demonstrate structural topology nearly identical to those found in HSF1 and HSF2 DBDs (Nakai et al., 1997; Xiao et al., 2020), which corresponds to their remarkably similar DNA-binding affinities in high-throughput studies of transcription factor binding (Jolma et al., 2013). Although HSF5 contains a putative DBD similar to that found in other HSFs, it binds to unique DNA motifs rather than canonical HSEs (Yoshimura et al., 2024). This specificity appears to be crucial for the role of HSF5 in regulating genes associated with chromatin organization during spermatogenesis (Yoshimura et al., 2024).

Trimerization of HSF1 and other HSF family members, which is essential for their transcriptional activity, occurs through interaction of leucine zipper (LZ) domains LZ1-3, formed by two HRs (HR-A and HR-B) of hydrophobic and charged amino acid residues that facilitate oligomerization (Gomez-Pastor et al., 2018; Littlefield and Nelson, 1999; Sorger and Nelson, 1989; Zuo et al., 1994). A third HR region (HR-C) in the carboxy-terminal end of the protein forms LZ4 and disrupts oligomerization of HSF1 through inhibitory intramolecular interactions (Farkas et al., 1998; Rabindran et al., 1993), inhibiting trimerization under non-stress conditions. HR-A/B domains [and the resultant three-dimensional (3D) protein structures LZ1-3] are highly conserved between HSF1, HSF2 and HSF4. However, HR-C/LZ4, while conserved between HSF1 and HSF2, is lacking in HSF4 (Fig. 1). The absence of an HR-C-like domain in HSF4 likely leads to a lack of regulatory inhibition and allows HSF4 to form stable trimers and to remain constitutively active in tissues in which it is expressed (Nakai et al., 1997). The constitutive trimerization of HSF4 likely contributes to its specific role in eye lens development rather than a broader stress response (Xiao et al., 2020). Although HSF5 lacks the HR domains found in other HSFs, it likely employs distinct mechanisms of oligomerization that allow it to exist as both monomers and higher-order oligomers, contributing to its specialized role in spermatogenesis (Yoshimura et al., 2024).

The critical transcriptional activation functions of HSFs are located in the carboxyl terminal domain in HSF1, HSF2 and HSF4 (Green et al., 1995; Nakai et al., 1997; Tanabe et al., 1999; Yuan et al., 1997; Zhu and Mivechi, 1999). The activation domain (AD) of HSF1 consists of multiple subdomains, including transcriptional activation domain (TAD)1 and TAD2, which are involved in transcriptional initiation, elongation and recruitment of chromatin remodeling complexes (Pessa et al., 2024; Sullivan et al., 2001). Despite similar positioning of the AD in HSF1 and HSF2, the amino acid sequence of these regions is less conserved than the amino acid sequence of their DBDs (Fig. 1). However, the molecular consequence of this deviation is not well characterized. Early studies using a GAL4 reporter system suggested that the AD of HSF1 has significantly reduced transcriptional activation potential compared to that of HSF1 in a hemin-treated K562 human erythroleukemia cells (Yoshima et al., 1998). To date, the direct transcriptional activation capacity of the TAD of HSF2 has not been rigorously assayed in a direct manner despite extensive profiling of HSF2 genome occupancy and transcriptional programs (Himanen et al., 2022; Smith et al., 2022; Vihervaara et al., 2013). However, human HSF2 is sufficient to restore thermotolerance and survival under heat shock when expressed in HSF-null yeast (in which yeast contain a single, essential HSF), suggesting that any reduced transcriptional activation capacity is, nonetheless, able to rescue perturbed proteostasis in heat stress (Liu et al., 1997). Future work should carefully elucidate the context specificity and functional sufficiency of the transcriptional activation potential of HSF2.

The TAD and activity of HSF4 and HSF5 remain areas of active research. Although HSF4 contains a C-terminal TAD, early domain-mapping studies using GAL4-luciferase reporters suggested that human HSF4 lacks transcriptional activation capacity (Nakai et al., 1997). Subsequent work revealed that *HSF4* undergoes alternative splicing, resulting in two HSF4 isoforms, HSF4A and HSF4B, which appear to have distinct consequences on transcription, either as activators or repressors, respectively (Merath et al., 2013; Tanabe et al., 1999). Importantly, there is significant variation in HSF4 expression even between cell types of the same species (human and mouse), supporting the need for future work to carefully dissect its cell type-specific functions (Syafruddin et al., 2021; Tanabe et al., 1999). Although the precise nature and role of any ADs in HSF5 are not well documented, recent studies show that HSF5 binds to promoter regions near transcription start sites, indicating that HSF5 likely has a mechanism for regulating gene expression tailored to its unique role in spermatogenesis (Yoshimura et al., 2024).

For the HSF family of transcription factors, highly evolutionarily conserved DBD architecture and DNA-binding preferences anchor predictions of shared functions. Oligomerization domains and TADs are most similar between HSF1 and HSF2, whereas HSF4 and HSF5 diverge, generating the evolutionary opportunity for unique mechanisms of regulation. Elucidating the contexts in which their shared ancestry predominates, as opposed to environments in which partitioned adaptations arise, will be exciting frontiers for future studies of HSF biology. Moreover, the conserved DNA sequences and corresponding functional protein domains of HSF family members form the substrate of remarkably complex layers of regulation, including both positive and negative feedback protein–protein interactions and extensive post-translational modifications that influence subcellular localization and oligomer formation, which are elaborated elsewhere (Alasady and Mendillo, 2024; Kmiecik and Mayer, 2022). Many of these regulatory mechanisms have been elucidated only for HSF1; thus, clarifying the extent to which they are conserved, diverge or constitute novel layers of regulation in other HSF family members will be an important domain of future research.

## HSF functions

HSF1 is the most well characterized of the family, owing to its ubiquitous expression and unique role as the master transcriptional regulator of the HSR (Akerfelt et al., 2010a; McMillan et al., 1998). In the absence of cellular stress, HSF1 is sequestered in the cytoplasm, where it is maintained in a monomeric, inactive state through interactions with chaperone complexes composed of HSP40, HSP70 and the cytosolic chaperonin T-complex protein 1 subunit alpha (TCP1) ring complex (Abravaya et al., 1992; Gomez-Pastor et al., 2018; Neef et al., 2014; Shi et al., 1998; Zheng et al., 2016). In response to stress, HSF1 translocates to the nucleus and adopts a DNA-binding and transcriptionally competent conformation through trimerization (Neudegger et al., 2016). Although HSF1 knockout mice exhibit prenatal mortality in many, but not all, genetic backgrounds (Xiao et al., 1999), demonstrating its essential role in extra-embryonic development and postnatal growth, viable knockouts have enabled study in diverse disease models. Surprisingly, although *Hsf1* knockout mice show impaired induction of HSPs under heat shock and other stress conditions, they can tolerate heat shock surprisingly well without immediate adverse effects (Neueder et al., 2017), perhaps owing to redundant orthogonal mechanisms of stress tolerance or robustness to environmental insult in higher eukaryotes. In adult mouse tissues, deficiency of HSF1 has adverse consequences under diverse physiologic stresses, including oxidative stress (Yan et al., 2002), response to infection (Murapa et al., 2011), and non-heat proteotoxic stress as in Huntington's disease (Gomez-Pastor et al., 2018; Neueder et al., 2017). Although these studies are limited to mouse models, they recapitulate biochemical and histologic properties of human disease, such as reduced levels of HSF1 observed in human brain tissue of patients with neurodegenerative diseases obtained post-mortem (Gomez-Pastor et al., 2017).

HSF2 was first identified in the human cancer cell line HeLa and shown to bind an HSE and induce transcription of target genes (Schuetz et al., 1991). Phylogenetic analyses of HSF evolution suggest that the *HSF2* gene appeared with the evolution of jawed vertebrates around 473 million years ago, with such early evolution contributing to their sequence homology (Cunningham et al., 2022; Fujimoto and Nakai, 2010; Hastbacka et al., 2025). Despite the structural similarities between HSF1 and HSF2, the primary functions of HSF2 are less clear. *Hsf2* knockout mice are viable and fertile with no severe phenotypic abnormalities, suggesting that HSF2 is not critical for normal development and fertility. However, this may be explained by compensatory mechanisms of other HSFs or pathways (McMillan et al., 2002). Initially, HSF2 was reported to have only a limited role in promoting the HSR despite its ability to bind consensus HSEs *in vitro* and *in vivo* in mouse and human cell line models (Jaeger et al., 2016; Mahat et al., 2016; Mathew et al., 2001; Sistonen et al., 1992, 1994). Although its contribution to heat stress appeared to be minimal, these studies demonstrated that HSF2 can activate HSP gene expression in response to hemin-induced differentiation of K562 erythroleukemia cells (Sistonen et al., 1992), which was dependent on its interplay with HSF1 (Ostling et al., 2007; Sistonen et al., 1994). Subsequent studies demonstrated a role for HSF2 function in spermatogenesis (Wang et al., 2004; Widlak and Vydra, 2017), in which it regulates the response to various proteotoxic stresses, including proteasome inhibition (Joutsen et al., 2020; Mathew et al., 1998), and ethanol-induced and febrile-range thermal stress (El Fatimy et al., 2014; Shinkawa et al., 2011).

Another example of the functional difference between HSF1 and HSF2 is their distinct capacity to restore viability in yeast depleted of their single essential HSF. Intriguingly, despite its limited role in heat stress, exogenous expression of human HSF2 (but not human HSF1) is sufficient to rescue the viability of such yeast (Liu et al., 1997). Disruption of the HSF1 carboxy-terminal regulatory domain, which maintains its inactive monomeric state, enables HSF1 to rescue yeast viability (Liu and Thiele, 1999). This result suggests that during evolution from the ancestral HSF, HSF1 gained a layer of regulation, whereas HSF2 preserved ancestral mechanisms of basal activation. Overall, the interaction between HSF1 and HSF2 and the regulation of their activities appear to be highly context dependent. This conclusion is further supported by the fact that the composition and post-translational modifications of heterotrimers formed when HSF1 and HSF2 bind to DNA (Ostling et al., 2007; Rabindran et al., 1993; Sandqvist et al., 2009; Smith et al., 2022) may distinguish the heat-responsive activity from the developmental functions of HSFs (Sandqvist et al., 2009). Much remains to be learned about how the stoichiometry of the active HSF heterocomplex (e.g. one HSF1 and two HSF2 molecules or vice versa) influences transcriptional output in specific cellular contexts.

The remaining members of the HSF family of proteins have been less well characterized. HSF4 contains a similar DBD, but deviates in its transcriptional activator domain position and sequence (Fig. 1) (Nakai et al., 1997). HSF4 is highly expressed in the eye lens, where it is required for growth and differentiation. Mutations in HSF4 result in cataract development in humans (Fujimoto et al., 2004, 2008; Xiao et al., 2020). Similarly, HSF4 knockout mice exhibit cataracts caused by the downregulation of lens-specific proteins, such as γS-crystallin and beaded filament proteins, thereby mimicking the phenotype (Gao et al., 2017; Shi et al., 2009).

HSF5 shares sequence similarity to the conserved HSF DBD, but the function of these proteins remains largely uncharacterized, owing at least in part to their tissue-specific expression (Gomez-Pastor et al., 2018). Knocking out HSF5 in mice does not result in overt phenotypes in somatic tissues, but it causes meiotic arrest during spermatogenesis, resulting in male infertility owing to abnormal pachynema and increased apoptotic spermatocytes (Yoshimura et al., 2024).

Together, the HSF family represents a clear example of a single protein's function being adapted to specific biochemical and cellular environments. The striking evolutionary conservation of core components of the original HSF is a testament to the fundamental role these proteins play in cellular processes, as well as human health and disease.

## HSFs in development

Beyond their role in thermal stress response, HSFs also serve critical functions in organismal development, which was first demonstrated by HSP-independent disrupted oogenesis and larval development upon knockout of the single *Hsf* present in *Drosophila melanogaster* (Jedlicka et al., 1997). Later, similar observations were made in *Caenorhabditis elegans* larval development upon *hsf-1* depletion (Li et al., 2016) and embryogenesis in *Hsf1* knockout mice (Xiao et al., 1999). Further investigations revealed that loss of HSF1 and HSF2 in some genetic backgrounds of mouse models has profound consequences on development, especially of the brain and reproductive tissues (Akerfelt et al., 2010a; Duchateau et al., 2020). The range of phenotypes observed in different model systems likely reflects functional redundancy, and more research is needed to understand the breadth of the biological activity of HSFs.

**Fig. 2.**
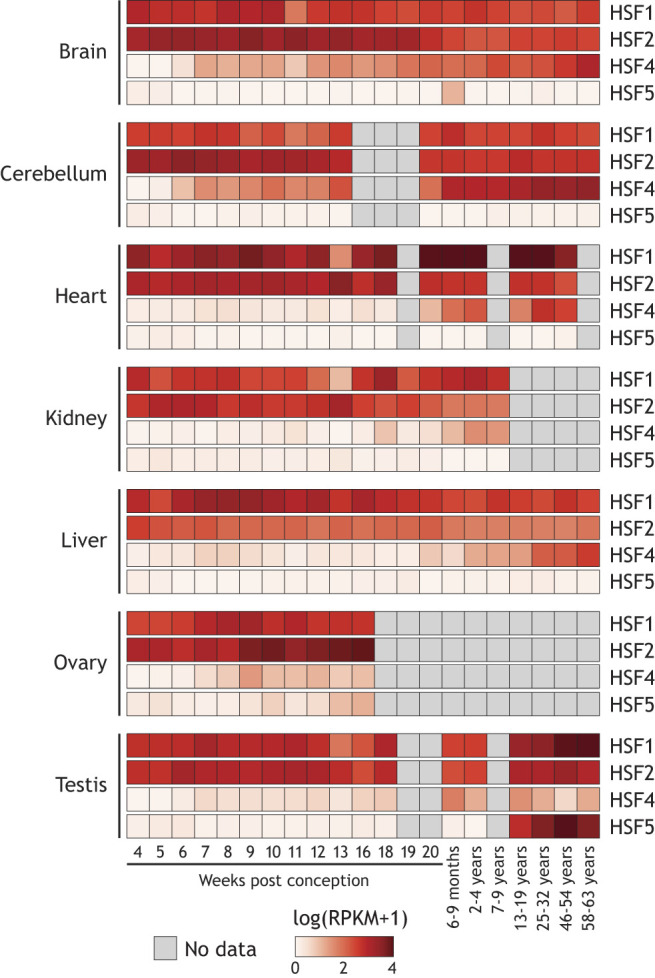
**Expression patterns of human HSF genes across tissues and developmental stages.** The chart displays HSF gene expression levels in multiple human tissues and at different developmental timepoints. Reads per kilobase of transcript per million mapped reads (RPKM) gene expression data for human samples for each tissue and developmental timepoint were downloaded from the Kaessmann Lab Shiny app for evo-devo mammalian organs (Cardoso-Moreira et al., 2019). Replicates ranged from one to four for each tissue and timepoint. For each gene, values represent the log normalized mean of replicate RPKM measurements+1.

A recent study revealed striking similarity in gene expression of *HSF1* and *HSF2* across tissues (including brain, cerebellum, heart, kidney, liver, ovary and testis) and developmental time beginning 4 weeks post conception and through adulthood (Fig. 2) (Cardoso-Moreira et al., 2019). Compared to the more ubiquitous expression of *HSF1* and *HSF2*, early prenatal *HSF4* expression is predominantly observed in the brain and cerebellum. HSF4 expression in other organs, including heart, kidney and liver, begins to increase after 6 months of development and persists during adult life (Duchateau et al., 2020; Syafruddin et al., 2021; Cardoso-Moreira et al., 2019), while *HSF5* expression is restricted to the testis and does not begin until around the time of puberty (Cardoso-Moreira et al., 2019). It is important to note the limitations of gene expression studies and emphasize the importance of work investigating the expression and localization patterns of HSF proteins in human tissues (Joutsen et al., 2024). Work by Joutsen et al. (2024) demonstrated that HSF1 protein exhibits nuclear expression in many epithelial cell types, whereas HSF2 expression is predominantly cytosolic in human tissues (Joutsen et al., 2024).

A number of studies have demonstrated a role for HSF1 and HSF2 as critical regulators of development in reproductive tissues. As Fig. 1 demonstrates, *HSF1* is ubiquitously expressed both prenatally and postnatally. Consistent with this, HSF1-deficient mice experience increased prenatal lethality, growth deficits and female infertility, despite select individuals surviving to adulthood (Xiao et al., 1999; Zhang et al., 2002). This phenotype is connected, in part, to HSF1 regulation of oocyte meiosis through HSP90α, (Metchat et al., 2009). Subsequent work revealed that mouse oocytes lacking HSF1 exhibited mitochondrial damage and increased susceptibility to oxidative stress, resulting in increased cell death (Bierkamp et al., 2010). *HSF1* is also expressed in the testis, which is highly thermosensitive (Fig. 2). Here, HSF1 plays a critical role in spermatocyte quality control (Izu et al., 2004). Intriguingly, heat stress of this tissue in mouse models and human cell lines does not induce a robust HSR, instead activating cellular apoptosis pathways via the pro-apoptotic *PMAIP1* (*NOXA*) gene (Izu et al., 2004; Janus et al., 2020). These findings serve as a stark example of cell type- and physiological context-specific HSF functions.

HSF2 is expressed at high levels in mouse testis, where it binds chromatin in genes responsible for sperm quality (Akerfelt et al., 2008; Sarge et al., 1994). Beyond elevated testis expression, spermatogonia are one of the few cell types in which HSF2 exhibits prominent nuclear expression (compared to cytosolic expression in most cell types) (Joutsen et al., 2024). In male mice, HSF2 knockout results in smaller testes, reduced sperm count and misshapen spermatid heads (Kallio et al., 2002; Wang et al., 2003). Dual knockout of *Hsf1* and *Hsf2* in male mice resulted in even more pronounced phenotypes, including the complete lack of mature sperm and resultant sterility (Wang et al., 2004). Further studies demonstrated the formation of HSF1–HSF2 heterotrimers in testes and their synergistic regulation of sex chromosomal multi-copy genes in post-meiotic round spermatids, providing mechanistic insights into more severe consequences of the double knockout (Akerfelt et al., 2010b; Sandqvist et al., 2009). In addition, HSF2 expression is important for ovary function. HSF2-deficient female mice, similarly to HSF1-deficient mice, suffer from oocyte meiotic defects, resulting in reduced ovarian follicles, abnormal eggs and hemorrhagic cystic follicles (Kallio et al., 2002).

Disruption of HSF1 and HSF2 function also has severe consequences for brain and central nervous system development in mice. Both HSF1 and HSF2 are expressed in prenatal and postnatal central nervous system tissues from mouse and human sources, while HSF4 exhibits very low prenatal expression followed by postnatal increases, particularly in the cerebellum (Duchateau et al., 2020). HSF1 loss results in enlarged ventricles, astrogliosis, accumulated ubiquitinated proteins and myelin loss (Homma et al., 2007; Santos and Saraiva, 2004). In addition, HSF1 loss leads to olfactory epithelium atrophy and olfactory sensory neurons death, when the cytoprotective role of HSF1 is absent (Takaki et al., 2006). Depletion of HSF2 disrupts neuronal migration, resulting in abnormal cortical lamination or layering of neurons in the cerebral cortex (Chang et al., 2006; El Fatimy et al., 2014; Kallio et al., 2002; Wang et al., 2003). These effects are mediated through the direct regulation of p35 by HSF2, which regulates cortical migration signaling (Akerfelt et al., 2010a; Chang et al., 2006). These data support a role for HSFs regulating genes beyond those encoding the canonical HSPs. Moreover, they elaborate a complex interplay between HSF1 and HSF2 in diverse cellular contexts to support proper organismal development and stress resilience.

## HSFs in neurodegenerative disease

A hallmark of neurodegenerative diseases is protein misfolding and aggregation. Not surprisingly, HSF1 and its target genes have a well-established link to neurodegenerative disease (Anckar and Sistonen, 2011). Although mutation or loss of HSF1 is not causative of these diseases, disruption of protein-folding machinery and chaperones exacerbates neurodegenerative phenotypes and disease progression (Gomez-Pastor et al., 2017, 2018). Reduced HSF1 activity and protein levels are observed in aging and in many protein folding-based neurodegenerative diseases, such as Huntington's, Parkinson's and Alzheimer's diseases, as well as amyotrophic lateral sclerosis (ALS) (Gomez-Pastor et al., 2018).

For example, in a mouse model of Huntington's disease, *Hsf1* knockout exacerbated the characteristic accumulation of mutant huntingtin protein and shortened the animal's lifespan (Hayashida et al., 2010). In human cell lines and mouse models, the expression of constitutively active HSF1 reduces huntingtin aggregate formation and improves the animal's lifespan (Fujimoto et al., 2005), rescuing the disease phenotype. In human cell and mouse models of Parkinson's disease, overexpression of α-synuclein (the aggregation of which in affected individuals leads to the hallmark findings of progressive loss of dopaminergic neurons in the substantia nigra, a midbrain region critical for coordinating movement) results in HSF1 depletion, further exacerbating disease progression (Kim et al., 2016). In the case of Alzheimer's disease [which is characterized by the accumulation of amyloid-β and loss of neurons in the hippocampus and cerebellum (Gomez-Pastor et al., 2018; Mavroudis et al., 2010)], reduced levels of HSF1 and chaperones in the cerebellum are observed in affected individuals and in mouse models (Jiang et al., 2013). Aggregates of another protein, TDP43 (also known as TARDBP), are considered a hallmark of ALS (Gomez-Pastor et al., 2018). *Hsf1* knockout mice exhibit ALS-like symptoms, including TDP43 accumulation and increased susceptibility to aggregation of other disease-related proteins (Chen et al., 2016). Importantly, in each of these mouse models of distinct neurodegenerative diseases, restoring HSF1 activity (either genetically, pharmacologically or by overexpressing the chaperone proteins to augment the protein folding milieu) has demonstrated promise in alleviating protein aggregation and neurodegenerative phenotypes (Fujimoto et al., 2005; Gomez-Pastor et al., 2017, 2018; Neef et al., 2010, 2011; Westerheide et al., 2004). These observations highlight the non-thermal stress functions of HSF proteins and their potential for restoring protein homeostasis in neurodegenerative diseases.

The discussed neurodegenerative diseases exhibit enhanced degradation of HSF1 during disease progression. In mouse models of Huntington's disease, elevated activity of casein kinase 2 (CK2) can promote the degradation of HSF1 through phosphorylation at Ser303 and Ser307 (Gomez-Pastor et al., 2017, 2018). Inhibition of CK2 led to stabilized HSF1 levels, increased chaperone gene expression and a concomitant reduction in huntingtin protein aggregation (Gomez-Pastor et al., 2017). CK2 levels are also elevated and correlate with disease progression in both clinical samples and mouse models of Parkinson's and Alzheimer's diseases, as well as ALS (Gomez-Pastor et al., 2018). Further mechanistic studies of CK2 activity, particularly in non-Huntington's neurodegenerative diseases, are needed to identify additional disease-contributing factors and to refine therapeutic strategies.

Studies of how other proteins in the HSF family are connected to neurodegenerative diseases are notably sparse. Knockout of HSF2 in mouse cell models of Huntington's disease revealed increased protein aggregation and reduced lifespan, similar to previously described results for HSF1 (Shinkawa et al., 2011). Elegant work by Homma et al. (2007) demonstrated that HSF1 knockout mice exhibit spinal cord demyelination, which progressed in an age-dependent manner. These findings were not observed for single HSF2 or HSF4 knockout mice; however, the demyelination and astrogliosis phenotypes were exacerbated with combination knockout of HSF1 and either HSF2 or HSF4 (Homma et al., 2007). These results demonstrate complex compensatory mechanisms between HSF family members in the brain, highlighting the importance of studying HSF interactions in physiology and disease.

Considering the shared DNA occupancy and co-regulation of chaperones by HSF1 and HSF2, together with expression of HSF2 (and HSF4) in brain tissues (particularly in the cerebellum, a region frequently affected in neurodegenerative disorders) (Carithers et al., 2015; Duchateau et al., 2020), each HSF family member likely plays unique and interdependent roles in the progression of neurodegenerative diseases. For example, in a study of HSF1 genome-wide activity in Huntington's disease, changes in expression of cytoskeletal and adhesion genes were observed, despite no changes in HSF1 DNA binding (Riva et al., 2012). Interestingly, recent reports have revealed a role for HSF2 in regulating cell–cell adhesion genes, which raises the intriguing possibility that HSF2 may contribute to the findings by Riva et al. (2012) in Huntington's disease models (Joutsen et al., 2020). These observations highlight the need for more comprehensive evaluation of HSFs in neurodegeneration, individually and in concert, which will inform understanding of HSF biology and potentially reveal new approaches for therapy to treat these diseases.

## HSFs in cancer

A substantial body of work has established HSF1 as a ‘non-oncogene addiction’ factor that supports tumor initiation and progression. Consistent with this, *Hsf1* knockout mice exhibit a decreased incidence of tumors when subjected to oncogenic stimuli in oncogenic, tumor suppressor and chemical models of carcinogenesis (Dai et al., 2012, 2007; Jin et al., 2011; Kourtis et al., 2018; Meng et al., 2010; Min et al., 2007; Xi et al., 2012). Although HSF1 does not cause cancer itself, its elevated activity allows rapidly proliferating cancer cells to address the heightened demand on protein quality control imposed by stresses, such as aneuploidy and nutrient restriction (Dai and Sampson, 2016; Luo et al., 2009; Solimini et al., 2007).

The different mechanisms of HSFs activation in tumors and normal cells, as well as the biological consequences of these altered activities, are only beginning to emerge. In cancer, in addition to promoting the gene expression of canonical HSP target genes, HSF1 also promotes expression of many non-canonical target genes with roles in diverse biological processes, including cell cycle regulation, translation, adhesion and metabolism (Alasady and Mendillo, 2020; Mendillo et al., 2012). Recent studies have shown that at least some of these targets contribute to proteostasis in surprising ways. For example, the bifunctional arginine demethylase and lysyl-hydroxylase JMJD6 (JMJD6) forms a positive-feedback loop with HSF1 to restore proteostasis (Alasady et al., 2024), while the mitochondrial ribosomal protein large ribosomal subunit protein uL18m (MRPL18) also functions in the cytosol to regulate translation of stress response proteins (Zhang et al., 2015), and phosphoglycerate kinase 1 (PGK1) has been implicated in the regulation of stress-induced autophagy (Qian et al., 2017), suggesting that other targets may, likewise, participate in proteostasis control.

Indeed, in breast, lung and colon cancers, high expression of HSF1 target genes (‘HSF1 cancer signature’) is associated with increased risk of metastasis and reduced survival (Carpenter and Gokmen-Polar, 2019; Mendillo et al., 2012). Tumors of diverse histopathological origin exhibit elevated levels and nuclear localization of HSF1 (Alasady and Mendillo, 2020; Dai and Sampson, 2016; Mendillo et al., 2012). Indeed, HSF1 activity can serve as a predictive biomarker for cancer outcomes, with elevated levels correlating with poor prognosis (Alasady and Mendillo, 2020; Bjork et al., 2018; Dai, 2018; Mendillo et al., 2012; Santagata et al., 2011).

The non-oncogene addiction role of HSF1 is evident in estrogen receptor alpha (ERα; also known as ESR1)^+^ breast cancer. Here, HSF1 hyperactivity upregulates HSP90, which chaperones the folding of ERα along with kinases and other proteins essential for rapid proliferation (Vydra et al., 2019; Whitesell et al., 2014; Xi et al., 2012). This elevated activity, in turn, potentially contributes to resistance to anti-estrogen therapies in breast cancer (Silveira et al., 2021). Recent work elucidated the ability of HSF1 to inhibit antitumor immune activity and CD8^+^ T-cell recruitment in breast cancer (Jacobs et al., 2024), establishing a link between cellular stress pathways and tumor–immune interactions. It follows that targeting these pathways could augment response of tumors to the rapidly developing field of immunotherapy.

Although HSF1 has been extensively investigated in human cancer tissues, information on HSF2 and HSF4 is mostly limited to mRNA expression datasets. These data demonstrate elevated expression of *HSF2* and *HSF4* in lung, breast, liver, esophageal and colorectal cancers (Meng et al., 2017; Puustinen and Sistonen, 2020; Zhong et al., 2016), but decreased expression of *HSF2* in prostate cancer (Bjork et al., 2016; Chen et al., 2022). However, protein expression and functional consequences of elevated expression of HSF2 and HSF4 in tumors require further investigation.

The impact of HSF1 on cancer biology extends beyond cancer cell-autonomous effects and is involved in supporting a tumor microenvironment permissive to cancer cell growth and metastasis. HSF1 is activated in essential for tumor microenvironment cancer-associated fibroblasts (CAFs), in which higher levels of HSF1, similarly to increased levels of HSF1 in tumor cells, correlate with poor prognosis (Scherz-Shouval et al., 2014). Consistent with this, depleting HSF1 from CAFs reduces xenograft tumor proliferation (Scherz-Shouval et al., 2014). These effects result from HSF1 directing a distinct, but complementary, transcriptional program to that previously defined in cancer cells themselves (Scherz-Shouval et al., 2014). Considering the pleiotropic roles of HSF1 in cancer cells, as well as the supporting stroma (Grunberg et al., 2021; Hockemeyer et al., 2024; Levi-Galibov et al., 2020; Shaashua et al., 2022), HSF1 may be a promising target for cancer therapy. Initial positive results from both direct and indirect targeting of HSF1 have been reported in mouse xenograft models of leukemia and prostate cancer (Dong et al., 2020; Santagata et al., 2013).

Unlike the consistently pro-tumorigenic role of HSF1, HSF2 and HSF4 functions in cancer are complex and context dependent, both promoting and suppressing various aspects of tumorigenesis. HSF2 demonstrates oncogenic activity across multiple cancer types through diverse mechanisms. In human hepatocellular carcinoma cell lines, HSF2 promotes proliferation by positively regulating aerobic glycolysis (Yang et al., 2019). Similarly, HSF2 is overexpressed in human lung cancer tissues and enhances proliferation and migration in normal lung epithelial and lung cancer cell lines (Zhong et al., 2016). In breast cancer, HSF2 promotes tumorigenesis through interaction with FAD-linked oxidoreductase ZEB1 (ZEB1), a transcription factor critical for promoting epithelial-to-mesenchymal transition, a hallmark of aggressive cancers (Li et al., 2014). Additionally, HSF2 exhibits pro-malignant functions in esophageal squamous cell cancer by suppressing apoptosis (Meng et al., 2017). However, in prostate cancer, HSF2 suppresses tumor progression by inhibiting pro-invasive phenotypes (Bjork et al., 2016). This apparent contradiction may be explained by previously unappreciated cell-type and disease-phase specificity of HSF2 functions. Recent work by Pessa et al. (2025) revealed that dynamic regulation of HSF2, but not HSF1, expression and its subcellular localization drives cell proliferation during preinvasive tumor expansion, while its downregulation by transforming growth factor-beta (TGF-β) becomes necessary for invasion and metastatic potential. The authors posit that HSF2 expression may be restored in the secondary tumor to promote growth and stress tolerance.

Similarly to HSF2, HSF4 also acts as both a tumor suppressor and an oncogene in a tissue-specific manner. In mouse models, HSF4 deficiency induced cellular senescence through upregulation of p21 and p27, suppressing spontaneous lymphoma/thymoma development in p53 (also known as *Trp53*)- or Arf-null (tumor suppressor genes) mice, while paradoxically increasing sarcoma incidence (Jin et al., 2012). Conversely, in human colorectal and renal cell carcinomas, HSF4 promotes tumor progression by activating c-MET (also known as MET; a receptor tyrosine kinase pathway with potent oncogenic activity) signaling and enhancing proliferation, migration and invasion (Zhang et al., 2023). The discussed studies focused on individual effects of HSF4 but did not investigate potential interactions or compensatory mechanisms with other HSF family members, leaving unexplored whether HSF1 or HSF2 might modulate the contrasting functions of HSF4 or whether their combined activities influence the observed tissue-specific outcomes.

Adding to this complexity, each of the discussed HSF family members has been reported to regulate hypoxia-inducible-factor-1α (HIF-1α) (Chen et al., 2011; Gabai et al., 2012), which increases vascular endothelial growth factor (VEGF), a potent signaling molecule for new blood vessel formation and a target of cancer therapies. Collectively, these data emphasize that the impact of HSFs on cancer progression – whether tumor suppressive or supportive of malignancy – is dictated by cellular context, disease stage and the regulatory landscape, underscoring the need for nuanced investigation of both individual and cooperative HSF functions in cancer.

These studies of HSF2 and HSF4, along with most work on HSF1 in cancer, have historically examined individual family members without considering their potential interplay. Yet, given their ability to bind similar consensus sequences and form hetero-oligomeric complexes, understanding cooperative and antagonistic interactions among HSFs is essential for elucidating their context-dependent roles in cancer (Roos-Mattjus and Sistonen, 2021). Existing studies highlight distinct regulatory modes among HSF family members and suggest that their differential expression at early stages does not prevent them from cooperating functionally at later stages.

In this regard, recent research has significantly advanced our understanding of the cooperative roles of HSF1 and HSF2 in cancer biology. Across diverse cancer types, HSF2, which was traditionally considered to have a limited role in the HSR, was found to physically and functionally interact with HSF1 (Smith et al., 2022). This interaction drives a shared transcriptional program encompassing canonical HSP genes and non-canonical targets involved in metabolism and proliferation. Consistent with these findings, unbiased coessentiality analyses (Amici et al., 2021) demonstrated that HSF2 is highly coessential with HSF1 across hundreds of cancer cell lines from diverse histopathological origins. Chromatin immunoprecipitation coupled with sequencing in aggressive breast and prostate cancer cell lines further revealed nearly identical chromatin-occupancy profiles for HSF1 and HSF2, spanning both canonical HSP genes and non-HSP targets critical for tumor growth. Loss of HSF1 caused a global reduction in HSF2 chromatin occupancy, indicating that HSF1 is required for efficient HSF2 binding – potentially through interrelated mechanisms governing HSF2 protein stability (Daupin et al., 2025; Santopolo et al., 2021).

Together, these findings support a robust functional interplay between HSF1 and HSF2 across a broad range of physiological conditions and raise the possibility that functions previously attributed solely to HSF1 may instead reflect coordinated activity with its paralogs. This emerging view underscores the importance of considering the full complement of HSFs, especially in physiologically relevant contexts of cellular stress.

## Drugging HSFs

Considering the critical roles that HSFs play across diverse physiological and pathological processes, their potential as therapeutic targets has attracted considerable interest (Carpenter and Gokmen-Polar, 2019; Dong et al., 2019; Neef et al., 2011; Sharma and Seo, 2018). Many reported small-molecule HSF inhibitors appear to work indirectly, for example, by disrupting HSF1-associated signaling networks or chaperone complexes, thereby reducing the oncogenic transcriptional programs that support tumor growth, therapy resistance and metastasis. Nonetheless, this rapidly growing area of investigation generates optimism for targeting the role of HSFs in disease.

KRIBB11 was one of the first identified small molecules to target HSF1. It has been shown to interfere with the recruitment of p-TEFb, a protein complex essential for transcriptional elongation, thus affecting HSF1 activity and suppressing HSP70 expression, which in turn inhibited cancer cell proliferation and tumor growth in xenograft models of colorectal cancer (Yoon et al., 2011). Another potentially direct HSF1 inhibitor, IHSF115, was developed following high-throughput screening efforts. IHSF115 dose dependently inhibits HSF1 transcriptional activity in cancer cell lines, in which it binds HSF1 DBD but does not disrupt HSF1 binding to DNA, as demonstrated by electrophoretic mobility shift assays and by chromatin immunoprecipitation followed by quantitative PCR (Vilaboa et al., 2017). Instead, the authors suggest that transcriptional inhibition occurs by IHSF115 disrupting the HSF1-ATF1 interaction (a complex suggested to be important for HSF1 transcriptional activation) (Takii et al., 2015; Vilaboa et al., 2017). However, this study does not address how specific the transcriptional disruption of IHSF115 is to HSF1 or whether other transcription factors that interact with cyclic AMP-dependent transcription factor ATF-1 (ATF1)/cyclic AMP-responsive element-binding protein (CREB) are also affected, thus limiting the interpretation of the exciting results the compound has in inhibiting cancer cell line growth (Vilaboa et al., 2017).

Dennis Thiele's group significantly contributed to the identification of small molecules that can modulate HSF1 activity. Their work demonstrated that targeting the HSF1 pathway can effectively reduce tumor growth and improve survival in prostate cancer cell culture and mouse models, with a direct targeted HSF1 inhibitor (DTHIB). DTHIB was identified through a differential scanning fluorimetry screen targeting the structurally ordered HSF1 DBD (amino acids 63-117), and it physically engages this domain with nanomolar affinity (Dong et al., 2020). Rather than directly blocking DNA binding, DTHIB selectively stimulates nuclear HSF1 degradation via a proteasome- and FBXW7-dependent pathway, thereby inhibiting the HSF1 cancer gene signature. This, in turn, leads to reduced tumor progression in therapy-resistant prostate cancer animal models, including castration-resistant prostate cancer xenograft and neuroendocrine prostate cancer syngeneic models (Dong et al., 2020). Notably, because the HSF1 DBD is highly conserved across HSF family members (Fig. 1), it remains possible that DTHIB or similar DBD-targeting molecules could also affect HSF2, HSF4 or HSF5, although these predictions will need to be directly tested in future experiments.

More recently, homoharringtonine (HHT), a plant-derived alkaloid, was shown to inhibit HSF1 transcriptional activity through high-throughput screening with an HSE-driven luciferase reporter (Li et al., 2024). Molecular docking studies suggest that HHT can bind HSF1 within the DBD (Li et al., 2024). Using human pancreatic cancer cell lines, the authors demonstrate that HHT significantly reduced HSF1 target gene transcription, inhibited cell viability in lines with high HSF1 expression by inducing apoptosis, and markedly reduced tumor progression in an orthotopic xenograft model, with greater potency than KRIBB11 or DTHIB (Li et al., 2024). However, these results are likely explained by the well-established role of HHT as a translation elongation inhibitor (Huang, 1975), in which inhibition of protein synthesis leads to a dominant transcriptional consequence of HSF1 inactivation (Alasady and Mendillo, 2020; Santagata et al., 2013).

In parallel, NXP800 (also known as CCT361814), a first-in-class, orally bioavailable small-molecule HSF1 pathway inhibitor, has advanced from preclinical development to clinical evaluation (Cheeseman et al., 2017). NXP800 was discovered through a cell-based phenotypic screen and inhibits HSF1-mediated transcription, leading to repression of HSP gene expression and activation of the integrated stress response (Cheeseman et al., 2017; Pasqua et al., 2023; Welti et al., 2025). Subsequent studies have clarified that the consequence on HSF1 activity occurs indirectly, namely through GCN2, an amino acid-sensing kinase that activates the integrated stress response (Welti et al., 2025). This compound has demonstrated antitumor activity in xenograft mouse models of ovarian cancer (Cheeseman et al., 2017), treatment-resistant prostate cancer (Welti et al., 2025) and osteosarcoma (Racineau et al., 2026). NXP800 is currently under evaluation in a phase 1a/1b clinical trial (NCT05226507), representing the first HSF1 pathway inhibitor to enter clinical testing (Pasqua et al., 2023).

Development of these compounds has progressed despite the inherent limitations of targeting transcription factors with significant stretches of intrinsically disordered regions, and they underscore the growing feasibility of pharmacologically modulating these challenging targets. However, an added complexity is that HSF1 is involved in essential cellular processes, and its complete inhibition could lead to undesirable side effects. Therefore, selective modulation of HSF1 activity, rather than complete inhibition, may be a more viable therapeutic strategy. Given the cooperative interaction between HSF1 and HSF2, targeting both factors simultaneously may provide a more effective therapeutic approach. This strategy could disrupt the transcriptional programs that support tumor growth and survival more comprehensively than targeting HSF1 alone. However, simultaneous targeting of multiple HSF family members introduces additional challenges: HSF2 also contributes to essential processes – including corticogenesis, spermatogenesis and proteostasis – in neurodegenerative disease, and the high conservation of the DBD across HSFs raises the possibility of off-target inhibition when using DBD-directed compounds. Thus, achieving isoform-selective modulation may require targeting domains outside the DBD or exploiting isoform-specific protein–protein interactions. Moreover, recent work demonstrates that targeting HSF4 enhances the efficacy of cabozantinib [a multi-target receptor tyrosine kinase inhibitor of VEGF receptor (VEGFR), c-MET and proto-oncogene tyrosine-protein kinase receptor Ret (RET)] and immune checkpoint inhibitors in renal cell carcinoma, with combination therapy showing synergistic effects in reducing cell proliferation and tumor growth (Saito et al., 2025). Although HSF4 and HSF5 are less well characterized and their druggability remains to be explored, their involvement in critical biological processes suggests potential therapeutic applications.

## Future directions for HSF biology

Several exciting areas of HSF biology remain underexplored, presenting opportunities for future research to deepen our understanding of these proteins’ functioning and their role in human health. Several groups, including ours, have used comprehensive genetic approaches, such as genome-wide chromatin occupancy for multiple HSF family members and whole transcriptome profiling, to supplement traditional single-gene studies and revealed additional layers of insight and nuanced relationships between HSFs. Functional genomic studies using CRISPR-based technologies empower more systematic and thorough interrogation of stress responses compared to single-gene knockout studies (Guo and Kampmann, 2022). Analytic strategies leveraging large-scale CRISPR screening datasets, such as coessentiality, have identified novel stress-response regulators with previously unappreciated consequences on cell fitness (Amici et al., 2021, 2022, 2023, 2024). Additionally, high-throughput chemical–genetic screens can further enhance our understanding by integrating chemical perturbations with genetic data, further expanding roles for stress response regulators and revealing pharmacological vulnerabilities that depend on the activation status of these factors (Brockway et al., 2020).

Future research should also integrate multi-omics approaches, combining genomics, transcriptomics, proteomics and metabolomics to study HSFs (Pessa et al., 2024). This holistic view can reveal how HSFs regulate cellular processes at multiple levels, from gene expression to protein function and metabolic pathways. Such integrative studies can uncover new layers of regulation and provide a more detailed understanding of HSF biology.

The study of these systems in physiologically relevant models is crucial. For instance, our work showed similarities in HSF1/2 function not with acute thermal stress but with non-thermal, growth-associated stresses (Smith et al., 2022). Applying this paradigm to HSF biology and other systems may reveal additional context-dependent protein functions. Moreover, understanding HSF biology in physiologically relevant models is essential for translating basic research findings into clinical applications. Many studies have used two-dimensional cell culture or simplified animal models that do not fully replicate the complex environments found in human tissues. In cancer, using 3D cellular models, such as spheroids and organoids, along with immunocompetent animal models, can provide a more accurate picture of how HSFs function *in vivo* (Grunberg et al., 2020; Hockaden et al., 2025; Williams et al., 2025). These models can help capture the cell nonautonomous and intercellular functions of stress response factors (van Oosten-Hawle and Morimoto, 2014), leading to a better understanding of how HSFs contribute to health and disease in a whole-organism context.

Advancing our understanding of HSF biology requires a multifaceted approach that includes comprehensive stress response studies, compact CRISPR-based functional genomics, physiologically relevant models, multi-omics integration and therapeutic targeting. These future directions hold the promise of uncovering new insights into HSF function and translating these findings into clinical applications that can improve human health.
